# Male breast cancer: a report of 25 cases

**DOI:** 10.11604/pamj.2020.37.343.23004

**Published:** 2020-12-15

**Authors:** Majdouline El Fouhi, Abdelhalim Mesfioui, Abdellatif Benider

**Affiliations:** 1 Laboratory of Genetic, Neuro-Endocrinology and Biotechnology, Ibn Tofail University, Kenitra, Morocco,; 2 Oncology-Radiotherapy, Mohammed VI Cancer Treatment Center, Ibn Rochd University Hospital, Casablanca, Morocco

**Keywords:** Breast cancer, male, clinical, pathological features, survival

## Abstract

Male breast cancer is a rare disease accounting for less than 1% of all breast cancer diagnoses worldwide to our knowledge. The aim of this retrospective study is to analyse the epidemiologic, clinical, therapeutic and evolutive profiles of this disease and to compare some cancer aspects between male and female in 25 cases collected at Mohamed VI Oncology Center at the University Hospital of Casablanca between 2012 and 2018. Of all breast cancers, men with breast cancer make up a minority. Male compared to female breast cancers occurred later in life with higher stage and more estrogen receptor-positive tumors. The median age was 67.7 years. The average diagnosis delay was 15.7 month. Cancer was discovered through self examination in 76.1% of cases. The mean diameter was 3.5 cm and range from 1-6 cm. According to the tumor-node-metastasis (TNM) classification, tumors were classified as T1-T2 (40%) and T3-T4 (60%). Infiltrating ductal carcinoma was the most frequent (92%) and 1 case of lobular carcinoma. Axillary nodal involvement was present in 82.4% of cases. Hormonal receptors were positive in 83% of cases. 86.6% of our cases present metastasis. Bone was the most representative site. Surgery was usually mastectomy with axillary clearance. It was possible to follow 21 of the patients. The median of follow-up was 12 months. The evolution has been characterized by local recurrence in 6 cases. There was 9 cases of death. Death was usually due to comorbid disease and to the advanced age. The 5 years overall survival rates were 57%.

## Introduction

Male breast cancer (MBC) is a rare type of cancer in the breast cancer series and in the male population. MBC incidence is generally low compared with the female breast cancer (FBC), the highest rates adjusted for age occur in Israel (0.08 per 100,000 person-years), while the rates are the lowest in Southeast Asia, particularly in Thailand (0.14 per 100000 person-years) [1]. In the region of Casablanca and according to cancer regestry, it has been count 45 cases of MBC versus 4794 cases of FBC between 2008 and 2012. The reason of the low incidence rate in men is the relatively low amount of breast tissue along with the difference in their hormonal environment. During the last few years, there has been an increase in the incidence of this disease, along with the increase in female breast cancer [2]. The genetic susceptibility for the population is the reason for this variability, genetic studies in males however, have shown that germline mutations in BRCA2 alone account for the majority of hereditary breast cancer [3,4]. Common factors of breast cancer risk, such as hormonal and environmental factors, are also involved in the pathogenesis of breast cancer. Male compared with female breast cancers occurred later in life with higher stage, and more estrogen receptor-positive tumors [5]. The management of breast cancer among men is generalized from the management of breast cancer in women [6,7]. Data usually interest female breast cancer (FBC) studies, limited information is available about the epidemiology, prognosis, quality of life and other data about MBC. In this review, we aim to report informations about the clinico-pathological characteristics, treatment and prognostic factors and the survival outcome of patients treated over six years in the university hospital of Casablanca-Morocco.

## Methods

A total of 25 cases of MBC were identified, study data were obtained from cancer registry of Casablanca, all males with pathologically confirmed invasive breast carcinoma diagnosed from 2012 to 2018 were included. Inclusion criteria were male patients with localized breast cancer, locally advanced or metastatic. We excluded from the study patients who had no follow-up after initial diagnosis. All the diagnosis of breast cancer had preoperative histological confirmation. Patients were evaluated for demographical characteristics, surgery type, clinicopathological characteristics. Demographic and tumor characteristics included : age at diagnosis, tumor size in centimeters, axillary lymph node status, stage, the average diagnosis delay, grade, histology, and estrogen receptor (ER) expression, treatement and evolution. An operating sheet was used to collect data, data analysis was performed using SPSS (21), and survival was calculated using the Kaplan Meier method.

## Results

**Figure 1:**
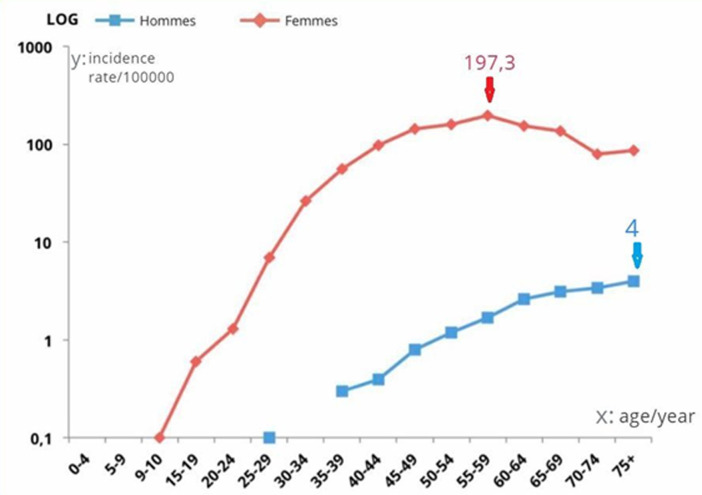
specific incidence rate (age/gender) of breast cancer, cancer registry of Casablanca 2008-2012

Twenty five patients at the oncology center of the university Hospital “Ibn rochd” in Casablanca, Morocco, with a diagnosis of breast cancer between January 2012 and December 2018 were retrospectively analyzed and evaluated in terms of epidemiological and histological characteristics and survival. The average age at time of diagnosis was 67.7 years (range: 36-87) which is more 10 years older than that noticed in women (Figure 1). Family history of breast cancer was observed in one case. The average diagnosis delay was 15.7 month. According to the TNM classification, tumors were classified as T1: 2 cases (8%), T2: 8 (32%), T3 and T4, 6 and 9 cases, respectively, 24% and 36%. The mean diameter was 3.5 cm and range from 1cm to 6cm. In 7 patients (28%), locally advanced disease was classified as N1, while in 10 of cases (40%) was classified as N2 and 8 (32%) was classified as N3. 86.6% of cases was presented with metastatic disease already, the most representative site of metastasis was the bone. According to the classification Scarff-Bloom-Richardson; 71.4% was classified grade II and 28.6% was classified grade III. Axillary nodal involvement was present in 82.4% of cases and lympho-vascular emboli was seen in 68.8%. Infiltrating ductal carcinoma was the most common subtype (92% of cases). One case of lobular carcinoma was seen. Less common subtypes included apocrine and papillary was observed. In addition, hormonal receptors were evaluated in 18 cases. Estrogen receptor (ER) and progesterone receptor (PR) were positive in 83% of cases (15 patients). Treatment was usually surgical. Complementary treatment included radiotherapy, chemotherapy and hormonotherapy. Surgery was usually mastectomy with axillary clearance or sentinel node biopsy. Radiotherapy (RT) was performed in 60% of our cases. Hormonal treatment (HT) was delivered in 66% of the cases. 83% of the patients have received chemotherapy (CT). We have followed 21 of the patients. The median of follow-up was 12 months. The evolution has been characterized by local recurrence in six cases. There were nine cases of death. Death was usually due to comorbid disease and to the advanced age. The 5 years overall survival rates were 57%.

## Discussion

Although breast cancer in men is far less common than breast cancer in women, it is associated with less favorable prognosis because diagnosis is usually made at an advanced stage. The average age of diagnosis in males is 60 years, which is ten years older than that noticed in female patients with the disease [8,9]. The average age of our patients (67.7 years) which is slightly higher than that seen in other series, we have noticed that the difference of the mean age at the diagnosis between males and females is superior to 10 years mentioned in the literature [8,9]. The most common clinical sign of breast cancer onset in men is a painless palpable swelling sub areolar. Other symptoms may include involvement nipple, with retraction and/or ulceration and/or bleeding [1]. 76.1% of our patients had descovered cancer trough self palpation. Collective reviews have shown predilection for the left side in a ratio of 1.07: 1. In our series we report also 52% of left desease side. Bilateral MBC has been reported in 1.9% patients [10]. We have seen one case of bilateral MBC in our series. About 90% of all male breast tumors have proved to be invasive ductal carcinomas, expressing high levels of hormone receptors with evident therapeutic returns. Infiltrating ductal carcinoma is the most predominant subtype with an incidence ranging from 64-93% [11,12]. Since the lobular system is not well developed in men, lobular carcinoma is uncommon, although, some cases have been reported in literature [13]. Our results match with this previous studies; 92% of our cases had an infiltrating ductal carcinoma and one case of lobular carcinoma. Axillary lymph node involvement is very common and clinically suspicious adenopathy has been seen in 40-55% patients. We report a rate of 82% of axillary nodal involvement, concerning females, several local studies have mentioned a rate between 50% and 65% of axillary lymph involvment [14-16]. The high rate in male is explained on the basis of lack of awareness and delayed diagnosis in males compared to females [17]. Breast cancer in males should be treated with the same strategy in women. The most common surgical procedure is modified radical mastectomy with axillary node dissection [18,19].

In our series, mastectomy with axillary clearance was the common surgical procedure combined with radiotherapy in men treated with mastectomy. Adjuvant radiation therapy has been shown to decrease local recurrence [20]. In a retrospective study of therapy in MBC, it was mentioned that the median survival of patients who underwent surgery alone was 33 months. However, for patients who received additional adjuvant therapy (chemotherapy) also radiation/hormones, either alone or in combination, the median survival rose to 86 months. Adjuvant therapy was most effective in large size, node positive and poorly differentiated tumours [21]. Frequently used chemotherapy regimens were cyclophosphamide methotrexate fluorouracil (CMF), fluorouracil epirubicin hydrochloride cyclophosphamide (FEC) and epirubicin cyclophosphamide (EC) [22]. The majority of our patients have recieved EC and FEC as adjuvant therapy. Due to the high positivity of ER/PR in MBC (83%). Most cases have recieved hormonotherapy (tamoxifen). Tamoxifen has been shown to lead to increased survival rates in women with hormone-sensitive disease and today is generally considered the standard adjuvant treatment for male breast cancer hormone-dependent [23]. Also hormonal therapy has been proven to help in metastatic disease in females and males [2,24,25]. The survival rate at 5 overall patient with male breast cancer is about 60% [26]. In our series, we report a five years overall survival rate of 57% which is less than seen in females. Some local previous studies interesting females have mentioned an overall survival rate at 3 years greater than 65% [14,27,28]. The number of histologically positive axillary lymph nodes and tumor size were significant prognostic factors. Another negative prognostic factor in our series is the advanced age at the time of diagnosis, as the increased presence of comorbidities may limit the possibility of treatment.

## Conclusion

Concerted efforts must be made to educate both the public and health professionals, in order to make earlier diagnosis and thereby improve prognosis. Earlier diagnosis and wide use of adjuvant treatments (RT/HT/CT) widely decreased local recurrences and increased survival rates in MBC, reaching female ones. Prognostic factors were also very similar to female ones.

### What is known about this topic

- The male breast cancer (MBC) is a rare and represents less than 1% of all malignancies in men and only 1% of all breast cancers incident;
- MBC occur later in life with higher stage and more estrogen receptor-positive tumors;
- The major problem is that breast cancer in men is often diagnosed later Survival rate in females is higher than seen in males.

### What this study adds

- Male breast cancer is rare, less data about this cancer is available, this study is a contribution;
- This study comes to determine the various epidemiological characteristics and survival outcome of male breast tumors in the university hospital in Casablanca;
- In this review, we provide some data about epidemiological characteristics of phyllodes that can serve clinicians.
